# The Role of Rhythm in Speech and Language Rehabilitation: The SEP Hypothesis

**DOI:** 10.3389/fnhum.2014.00777

**Published:** 2014-10-13

**Authors:** Shinya Fujii, Catherine Y. Wan

**Affiliations:** ^1^Heart and Stroke Foundation Canadian Partnership for Stroke Recovery, Sunnybrook Research Institute, Toronto, ON, Canada; ^2^Department of Radiology, Boston Children’s Hospital, Harvard Medical School, Boston, MA, USA

**Keywords:** rhythm, speech, language, rehabilitation, the SEP hypothesis

## Abstract

For thousands of years, human beings have engaged in rhythmic activities such as drumming, dancing, and singing. Rhythm can be a powerful medium to stimulate communication and social interactions, due to the strong sensorimotor coupling. For example, the mere presence of an underlying beat or pulse can result in spontaneous motor responses such as hand clapping, foot stepping, and rhythmic vocalizations. Examining the relationship between rhythm and speech is fundamental not only to our understanding of the origins of human communication but also in the treatment of neurological disorders. In this paper, we explore whether rhythm has therapeutic potential for promoting recovery from speech and language dysfunctions. Although clinical studies are limited to date, existing experimental evidence demonstrates rich rhythmic organization in both music and language, as well as overlapping brain networks that are crucial in the design of rehabilitation approaches. Here, we propose the “SEP” hypothesis, which postulates that (1) “sound envelope processing” and (2) “synchronization and entrainment to pulse” may help stimulate brain networks that underlie human communication. Ultimately, we hope that the SEP hypothesis will provide a useful framework for facilitating rhythm-based research in various patient populations.

## Introduction

Human beings have universally engaged in rhythmic musical activities such as drumming, dancing, singing, and playing musical instruments since ancient times (e.g., Mithen, 2005; Fitch, 2006; Conard et al., 2009). The presence of rhythmic sounds in the environment can result in spontaneous motor responses such as tapping, clapping, stepping, dancing, and singing (e.g., Kirschner and Tomasello, 2009; Sevdalis and Keller, 2010; Fujii et al., 2014). Rhythm serves as a potent catalyst to elicit positive affect (e.g., Zentner and Eerola, 2010), co-operation (e.g., Reddish et al., 2013), and social bonding (e.g., Freeman, 2000). In recent years, researchers have begun to explore the therapeutic potential of rhythm in speech and language rehabilitation. In this paper, we discuss the importance of rhythm as a medium of human communication and social interaction. Specifically, we discuss the important role of rhythm in speech perception and production, and summarize the relevant neuroscience literature. In addition, we present the “SEP” hypothesis, which postulates that (1) sound envelope processing and (2) synchronization and entrainment to a pulse, may help stimulate brain networks that underlie human communication. Finally, we provide examples of speech and language disorders [Parkinson’s disease, stuttering, aphasia, and autism] that can potentially benefit from rhythm-based therapy.

## Rhythm as a Medium of Communication

Rhythm, or the temporal organization of perceived or produced events, mediates communication and social interaction. For example, normal rhythm or rate of syllable production during speech is typically three to eight syllables per second (3–8 Hz) across many languages (Malecot et al., 1972; Crystal and House, 1982; Greenberg et al., 2003; Chandrasekaran et al., 2009). This rate range corresponds to natural movement frequencies of articulators including tongue, palate, cheek, jaw, and lips coupled with voicing (Peelle and Davis, 2012). If the rate is faster than 8 Hz, however, speech intelligibility is significantly reduced (e.g., Ahissar et al., 2001), suggesting that our brain may be “tuned” to the natural rhythm of vocal production.

Primate studies have shown a similar rhythmic tuning to communicative gestures such as lip-smacking (Fitch, 2013; Ghazanfar et al., 2013; Ghazanfar and Takahashi, 2014). Lip-smacking is often directed at another animal during face-to-face interactions, and is characterized by regular cycles of vertical jaw movement, often involving a parting of the lips (e.g., Ghazanfar et al., 2012). For example, Ghazanfar et al. (2013) used three types of video clips as visual stimuli in which monkey avatars were lip-smacking at frequencies of 3, 6, and 10 Hz. Interestingly, the preferential viewing times were significantly longer for the smacking rate of 6 Hz, which corresponds to the natural syllable production rate, compared with those of 3 and 10 Hz. Moreover, the monkeys in the study responded to the avatars in the video with their own rhythmic lip-smacking expressions, as if they were communicating with real monkeys. Based on these observations, Ghazanfar et al. (2013) suggested that monkey lip-smacking and human speech rhythms share a similar sensorimotor mechanism, and that human speech might have evolved from the rhythmic gestures or motor actions normally produced by our primate ancestors.

The idea that rhythmic motor actions mediate communication is supported by another primate study, which showed that rhythmic non-vocal sounds created by drumming actions served as communicative signals (Remedios et al., 2009). The mean rate of drumming actions by the macaque monkeys were around five beats per second (Remedios et al., 2009), which also corresponded to the natural syllable production rate during speech. Further investigation with functional magnetic resonance imaging (fMRI) showed that neural responses when animals listened to rhythmic drumming sounds overlapped with those when they listened to vocalizations (Remedios et al., 2009).

Human studies have also shown the importance of gestures and rhythmic motor actions for communication and social interaction. For example, lip movements affect the way in which people perceive speech syllables (McGurk and MacDonald, 1976). In congenitally blind individuals, verbal communication is often accompanied by hand gestures, although they have never seen hand gestures, and the listener cannot see the speaker’s movements (Iverson and Goldin-Meadow, 1998). Developmental studies have shown that newborn infants imitate adult facial and manual gestures (e.g., Meltzoff and Moore, 1977) and synchronize body movements with the articulated structure of adult speech (e.g., Condon and Sander, 1974). Furthermore, 3- to 4-month-old infants show altered vocalizations and synchronized limb movements in response to rhythmic dance music (Fujii et al., 2014), while 5- to 24-month-old infants engage in more rhythmic limb movements and smile more during music listening (Zentner and Eerola, 2010). Older preschool children spontaneously play the drum in synchrony when a human adult partner plays the drum (Kirschner and Tomasello, 2009). Thus, rhythmic sounds and motor actions are fundamental for communication and social interaction throughout development.

## The Role of Rhythm in Speech

**Figure 1:**
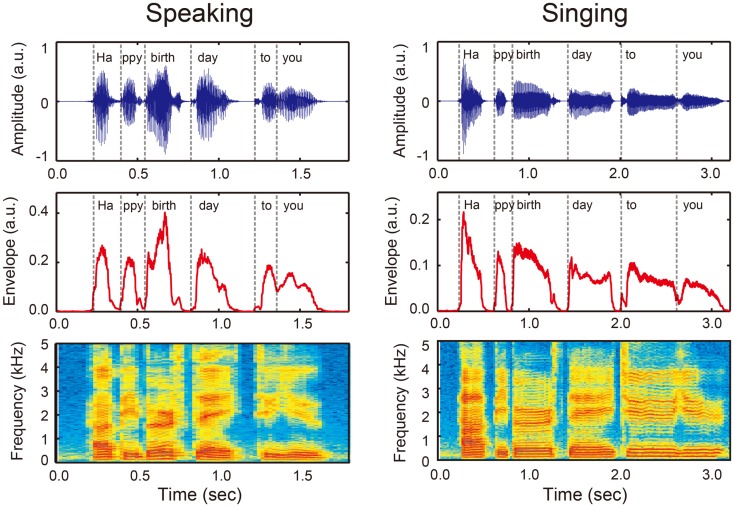
**An example of sound wave (upper panels), amplitude envelope (middle panels), and power spectrum (bottom panels) when a person speaks (left) and sings (right) “Happy birthday to you” that can be divided into six syllables (i.e., Ha/ppy/birth/day/to/you, see vertical dashed lines)**. Sound envelope is an important acoustic information that conveys temporal organization of phonemes and syllables or rhythm in vocalization. Note that rhythm in singing (right) has a more salient pulse- or beat-based timing compared with rhythm in speech (left).

Rhythm is essential to the understanding of speech. In order to comprehend spoken language, listeners are required to perceive temporal organization of phonemes, syllables, words, and phrases from an ongoing speech stream (Kotz and Schwartze, 2010; Patel, 2011; Peelle and Davis, 2012). An important source of acoustic information that conveys rhythm in speech is the *sound envelope*, which is defined as the acoustic power summed across all frequencies for a given frequency range (Kotz and Schwartze, 2010; Patel, 2011; Peelle and Davis, 2012). As illustrated in Figure 1, the phrase “Happy birthday to you” can be broken down into six syllables (i.e., “Ha/ppy/birth/day/to/you”), and these boundaries correspond to the pattern of the sound envelope (denoted by vertical dashed lines). Thus, burst patterns of the sound envelope represent rhythm or temporal organization in vocalization.

A number of studies have demonstrated the importance of rhythm or sound envelope in speech comprehension (e.g., Drullman et al., 1994a,b; Nazzi et al., 1998; Ahissar et al., 2001; Smith et al., 2002; Elliott and Theunissen, 2009; Bertoncini et al., 2011). For example, Shannon et al. (1995) tested the importance of rhythm in speech by minimizing the fine spectral information while preserving the sound envelope. Near perfect speech recognition performance was observed when individuals were presented with these speech stimuli. Consistent with this finding, smearing of the rhythm or sound envelope in speech sounds significantly reduced sentence intelligibility (e.g., Drullman et al., 1994a,b; Elliott and Theunissen, 2009). The reliance on rhythm or sound envelope to discriminate speech sounds has also been reported in infants (Nazzi et al., 1998; Bertoncini et al., 2011). Smith et al. (2002) further investigated the different roles of envelope and fine spectral structure in human auditory perception. They created sound stimuli called “auditory chimeras,” which consisted of the envelope of one sound and the fine spectral structure of another (Smith et al., 2002). Interestingly, when the two features (i.e., envelope and fine spectral structure) were in conflict, the pitch and location of sounds were determined by the fine spectral structure, while the words identified were based on the envelope (Smith et al., 2002). Thus, along with fine spectral information, sound envelope or rhythm is essential for speech intelligibility.

## Neural Correlates of Rhythmic Speech Perception

**Figure 2:**
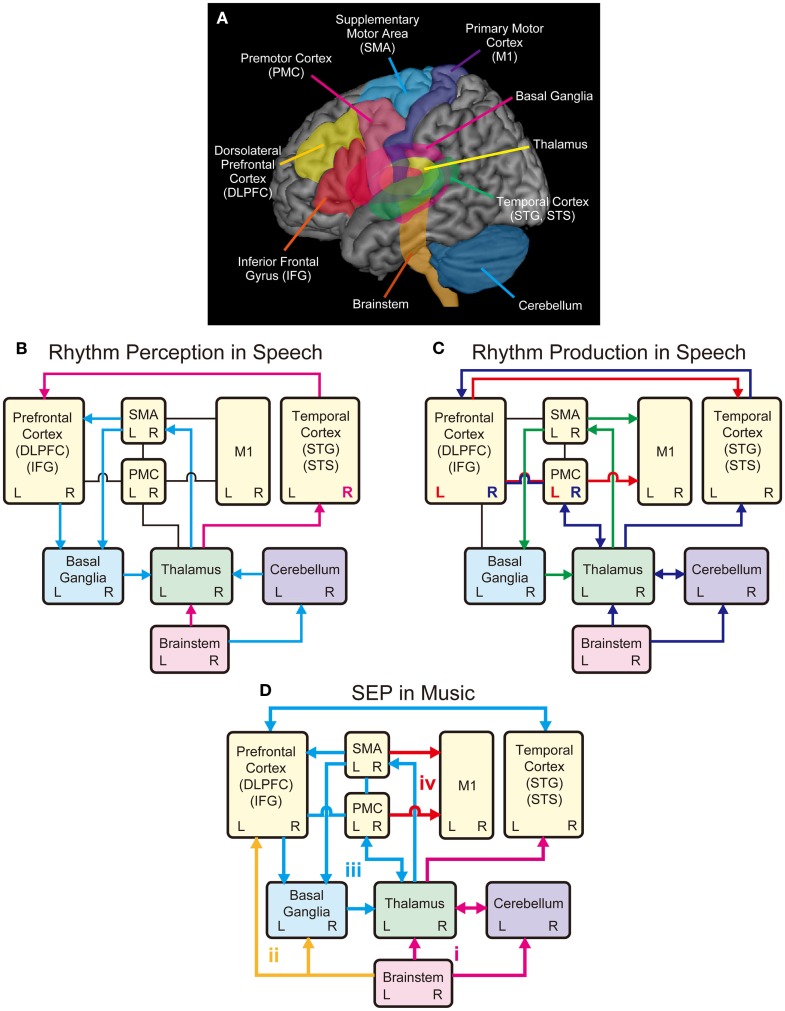
**Schematic models of shared brain network for rhythm perception and production in speech and music**. **(A)** Possible shared brain regions for rhythm processing in music and speech. **(B)** A model for rhythm perception in speech. The temporal cortex receives auditory inputs from the brainstem via the thalamus, which further transmits information to the prefrontal cortex (pink arrows). Processing of sound envelope or low-frequency temporal feature in the acoustic signals may be lateralized to the right hemisphere in the temporal cortex (see pink R). The cerebellum also receives the auditory inputs from the brainstem and relays information to the SMA via the thalamus, and further transmits information to the prefrontal cortex to process temporal events. The SMA and the prefrontal cortex transmit information to the basal ganglia (BG), which transmits information back to the cortex via the thalamus forming the BG-thalamo-cortical loop (light blue arrows). **(C)** A model for rhythm production in speech. The M1 receives inputs from the SMA, which forms the SMA-BG-thalamo loop, for rhythmic speech production (green arrows). The M1 also receives inputs from the left PMC and IFG, which transform speech sounds into motor commands (see red arrow and L). The left PMC and IFG also transmit information to the temporal cortex, which is associated with sensory predictions. The temporal cortex monitors the sensory predictions and the auditory feedback received from the brainstem via the thalamus. The feedback errors from the temporal cortex are sent to right PMC and IFG, which is interconnected with the thalamus and the cerebellum (see blue arrows and R). **(D)** A model for Sound Envelope Processing (SEP) and Synchronization and Entrainment to a Pulse (SEP) in music. Rhythm-based therapy may help stimulate brain networks involving (i) the auditory-afferent circuit consisting of brainstem, thalamus, cerebellum, and temporal cortex (pink arrows) for precise encoding of sound envelope and temporal events; (ii) the subcortical-prefrontal circuit for emotional and reward-related processing (yellow arrows); (iii) the BG-thalamo-cortical circuit for processing beat-based timing (light blue arrows); and (iv) the cortical-motor efferent circuit for motor output (red arrows).

A question arises then, regarding how rhythm or sound envelope is processed in the brain. fMRI studies have shown that the processing of sound envelope or low-frequency temporal feature in the acoustic signal is associated with activities in the inferior colliculus of the brainstem, the medial geniculate body of the thalamus, the Heschl’s gyrus (HG), the superior temporal gyrus (STG), and the superior temporal sulcus (STS) (Giraud et al., 2000; Boemio et al., 2005). The “asymmetric sampling in time (AST)” hypothesis postulates that low-frequency temporal features in the acoustic signals are lateralized to the right hemisphere, whereas high-frequency fine spectral features of the acoustic signals are lateralized to the left hemisphere (Poeppel, 2003; McGettigan and Scott, 2012). Consistent with this hypothesis, electroencephalography (EEG) studies have also shown that sound envelope processing is right lateralized (Abrams et al., 2008, 2009). Similarly, phase pattern of theta band (4–8 Hz) responses recorded from the temporal cortex using magnetoencephalography (MEG), especially in the right hemisphere, is correlated with the degree of speech intelligibility (Luo and Poeppel, 2007). Thus, the neural mechanisms underlying rhythm or sound envelope processing are likely to involve the brainstem, the thalamus, and the auditory regions in the temporal cortex, which may be lateralized to right hemisphere (see pink arrows and “R” in Figure 2B).

In parallel with the brainstem-thalamo-cortical (temporal auditory regions) pathway, there is another possible pathway for rhythm perception in speech, which involves the brainstem, the cerebellum, the thalamus, the supplementary motor area (SMA), the basal ganglia (BG), and the prefrontal cortex (light blue arrows in Figure 2B) [see Kotz and Schwartze (2010)]. Here, early auditory input is transmitted to the cerebellum via the cochlear nuclei of the brainstem (Huang et al., 1982; Wang et al., 1991; Xi et al., 1994; Kotz and Schwartze, 2010). The cerebellum is responsible for the encoding of event-based temporal structure, and relays information to the SMA via the thalamus, which further transmits information to the prefrontal cortex (Kotz and Schwartze, 2010). The SMA and the prefrontal cortex transmit temporal information to the BG, which transmits information back to the cortex via the thalamus forming the BG-thalamo-cortical loop (Kotz et al., 2009; Kotz and Schwartze, 2010, 2011). This closed-loop circuit is assumed to have functions to continuously evaluate temporal relations, extract temporal regularity, and engage in sequencing of temporal events and analysis of hierarchical structure (Kotz et al., 2009; Kotz and Schwartze, 2010, 2011; Schwartze et al., 2011). For example, when we listen to the six syllables of “ha/ppy/birth/day/to/you,” they are grouped into four words “happy/birthday/to/you” and perceived as one phrase (Figure 1). Thus, rhythm perception in speech can be regarded as finding the hierarchical structure of temporal events (Kotz et al., 2009; Kotz and Schwartze, 2010; Przybylski et al., 2013). The prefrontal cortex integrates information from the temporal cortex with that being processed in the BG-thalamo-SMA loop circuit to optimize speech comprehension (Kotz and Schwartze, 2010). Taken together, the sound envelope or rhythm is processed in the cortical and subcortical auditory-motor systems.

## Neural Correlates of Rhythmic Speech Production

In the previous section, we described the neural correlates of sound envelope or rhythm processing in speech perception. We now consider the neural correlates of rhythmic speech production. The directions into velocities of articulators (DIVA) model provide a useful framework to consider the neural mechanisms (see Guenther et al., 2006; Bohland et al., 2010; Tourville and Guenther, 2011). According to the DIVA model, rhythmic speech production is achieved by sequential control of the velocities of articulators including the upper and lower lips, the jaw, the tongue, and the larynx. In this model, the bilateral ventral primary motor cortex (vM1), which corresponds to the cortical homunculus of speech articulators (Penfield and Rasmussen, 1950; Penfield and Roberts, 1959), is responsible for outputting motor commands to the muscles of articulators [see also Kalaska et al. (1989), Ludlow (2005), Brown et al. (2008), and Olthoff et al. (2008)]. The vM1 receives inputs from the SMA connected with the BG and the thalamus (green arrows in Figure 2C) [see also Jurgens (1984) and Luppino et al. (1993)]. This is supported by fMRI studies that showed bilateral activities in the BG, thalamus, and SMA during speech production (e.g., Bohland and Guenther, 2006; Tourville et al., 2008). The BG-thalamo-SMA circuit is hypothesized to play a role in sequencing and self-initiation of speech, or rhythmic speech production in the DIVA model. This is based on clinical studies that showed speech production problems including involuntary vocalizations, echolalia, lack of prosody, stuttering-like output, variable rate, and difficulties with complex speech sequences following the impairment of the BG-thalamo-SMA circuit (e.g., Jonas, 1981; Ziegler et al., 1997; Ho et al., 1998; Pickett et al., 1998; Vargha-Khadem et al., 1998; Pai, 1999; Watkins et al., 2002).

In the DIVA model, the bilateral vM1 also receives inputs from left ventral premotor cortex (vPMC) and adjacent posterior inferior frontal gyrus (pIFG) (see red arrow and “L” in Figure 2C). These areas (i.e., left vPMC and pIFG) are hypothesized to form the “speech sound map,” which transform speech sounds (e.g., phonemes and syllables) into motor commands (Guenther et al., 2006; Bohland et al., 2010; Tourville and Guenther, 2011). In other words, the speech sound map is “mental syllabary” or repository of learned speech motor program [see also Levelt and Wheeldon (1994) and Levelt et al. (1999)]. The speech sound map has anatomical correspondence with Broca’s area (e.g., Dronkers et al., 2007) and has functional correspondence with the “mirror-neuron system” (Rizzolatti et al., 1996; Kohler et al., 2002). Language function is generally regarded as left lateralized because impairments of these brain regions (i.e., left vPMC and pIFG) lead to significant deficit of speech production (e.g., Dronkers, 1996; Kent and Tjaden, 1997; Hillis et al., 2004; Duffy, 2005).

The speech sound map (i.e., left vPMC and pIFG) is hypothesized to project not only to the vM1 to form the motor commands but also to the bilateral auditory areas in the temporal cortex (Guenther et al., 2006; Tourville and Guenther, 2011) (see red arrow in Figure 2C). The auditory areas include two locations along the posterior superior temporal gyrus (pSTG): the lateral one near the STS, and the medial one at the junction of the temporal and parietal lobes deep in the sylvian fissure (Guenther et al., 2006; Tourville and Guenther, 2011). These auditory areas are activated not only during speech perception but also during speech production (e.g., Buchsbaum et al., 2001; Hickok and Poeppel, 2004). The projections from the speech sound map to these auditory areas are responsible for predicting the sound being produced, which is compared with the actual auditory feedback being processed in the HG and the adjacent anterior planum temporale (PT) (Guenther et al., 2006; Tourville and Guenther, 2011). If the auditory feedback does not fall within the predicted range, the error signals are sent back to the right vPMC and pIFG according to the DIVA model (Guenther et al., 2006; Tourville and Guenther, 2011) (see blue arrows and “R” in Figure 2C). The right vPMC and pIFG are interconnected with the cerebellum via the thalamus, forming the “feedback control map,” which transforms the sensory error signals into corrective motor commands (Tourville and Guenther, 2011). This is based on fMRI studies that showed increased hemodynamic responses in the right PMC, pIFG, and the cerebellum during speech production under perturbed auditory feedback conditions (Tourville et al., 2008). Taken together, it is assumed that the BG-thalamo-cortical (vM1 and SMA) circuit is essential for rhythmic speech production, and the other areas (vPMC, pIFG, temporal cortex, and cerebellum) also play important roles for sensorimotor transformation and integration processes.

## Theoretical Rationale Underlying the Role of Rhythm for Speech and Language Rehabilitation: The “OPERA” and “SEP” Hypotheses

The aim of this section is to provide a rationale underlying the role of rhythm for speech and language rehabilitation considering the above mentioned neural correlates of rhythm perception and production in speech. Here, we present the “SEP” hypothesis, which is a rhythm-specific extension of the “OPERA” hypothesis (Patel, 2011, 2012, 2014), to explain how and why musical rhythm can benefit speech and language rehabilitation.

The OPERA hypothesis is a conceptual framework that postulates how general musical activities can facilitate speech and language processing (Patel, 2011, 2012, 2014). The OPERA hypothesis assumes that five conditions are needed to drive the benefit of musical activities for speech and language processing: (1) overlap: there is anatomical overlap in the brain networks during the processing music and speech, (2) precision: music places higher demands on these shared networks than does speech, in terms of the precision of processing, (3) emotion: musical activities that engage this network elicit strong positive emotion, (4) repetition: musical activities that engage this network are frequently repeated, and (5) attention: musical activities that engage this network are associated with focused attention. In other words, condition (1) describes shared neural underpinnings for both music and speech activities while (2)–(5) are distinctions of musical activities that may drive the neural plasticity enhancing the abilities of speech and language processing.

While the OPERA hypothesis provides a useful conceptual framework for general musical processing, it has a number of limitations that preclude its application to rhythm-based therapy. First, the OPERA hypothesis describes the possible overlap in brain networks during music and speech processing, but the explanation is restricted to perception (i.e., afferent process from cochlea to auditory cortex level). Within the context of rhythm, however, it is important to clarify the overlapping brain networks during sensorimotor coupling and production (i.e., cortical auditory-motor interaction and efferent process from the motor cortex to the spinal cord). Second, the OPERA hypothesis covers many aspects of general musical processing that include pitch and timbre. Here, we specifically discuss how rhythm itself meets with the five conditions of the OPERA hypothesis (i.e., overlap, precision, emotion, repetition, and attention). Third, under the OPERA hypothesis, it is not clear whether rhythm *per se* would have therapeutic potential in patient populations.

In order to address the above limitations, we propose the “SEP” hypothesis, which postulates two additional components to describe how and why rhythm in particular can be beneficial for speech and language rehabilitation: (1) sound envelope processing and (2) synchronization and entrainment to a pulse. The first key component of the SEP hypothesis, sound envelope processing, is adapted from the OPERA hypothesis (Patel, 2011, 2012, 2014), which postulates the major sources of overlap in the brain networks during rhythm perception in music and speech [see also Peretz and Coltheart (2003), Corriveau and Goswami (2009), Kotz and Schwartze (2010), and Goswami (2011)]. The second key component in the SEP hypothesis, synchronization and entrainment to a pulse, postulates the major sources of overlap in the brain networks not only for rhythm perception but also for rhythm production and sensorimotor coupling in music and speech (Guenther et al., 2006; Kotz et al., 2009; Bohland et al., 2010; Kotz and Schwartze, 2010, 2011; Tourville and Guenther, 2011).

In the SEP hypothesis, we assume that the overlap in brain networks for rhythm processing between speech and music involve the brainstem, cerebellum, thalamus, BG, M1, SMA, PMC, prefrontal cortex (DLPFC and IFG), and temporal cortex (STG and STS). As illustrated in Figure 2D, we propose four key circuits in the shared brain network to explain the potential benefit of rhythm-based therapy: (a) the auditory afferent circuit consisted of brainstem, thalamus, cerebellum, and temporal cortex (pink arrows) for precise encoding of sound envelope and temporal events; (b) the subcortical–prefrontal circuit for emotional and reward-related processing (yellow arrows); (c) the BG-thalamo-cortical circuit for processing beat-based timing (light blue arrows); and (d) the cortical motor efferent circuit for motor output (red arrows).

### Auditory afferent circuit

According to the OPERA hypothesis, musical activities place high demands on precise encoding of acoustic features including the sound envelope (Patel, 2011, 2012, 2014). This notion is supported by neuroimaging studies that showed more precise encoding of sounds in the brainstem of musicians compared with non-musicians (e.g., Musacchia et al., 2007, 2008; Parbery-Clark et al., 2011, 2012; Strait and Kraus, 2014; Strait et al., 2014). Interestingly, individuals with more musical training exhibit better encoding of speech sounds in the brainstem, larger cortical responses, and better speech sound perception (Strait and Kraus, 2014; Strait et al., 2014). Although the role of the cerebellum for speech and music perception is not mentioned in the OPERA hypothesis, we also include the cerebellum as a part of the auditory afferent circuit because it receives input from the cochlear nuclei (Huang et al., 1982; Wang et al., 1991; Xi et al., 1994; Kotz and Schwartze, 2010) and plays an important role in encoding the absolute duration of time intervals in successive acoustic events (Kotz and Schwartze, 2010; Teki et al., 2011a,b). For example, a recent neuroimaging study showed that perception of changes in musical rhythm (so-called “groove”) with drum sounds is associated with activity in the cerebellum and the STG (Danielsen et al., 2014). In addition, musicians showed enhanced activity in the cerebellum compared to non-musicians during temporal perception (Lu et al., 2014) and have larger volumes of cerebellum than non-musicians (Hutchinson et al., 2003). Taken together, we postulate that musical rhythm perception or sound envelope processing in music places high demand on the auditory afferent circuit consisted of the brainstem, thalamus, cerebellum, and temporal cortex (pink arrows in Figure 2D). This corresponds to the second condition of the OPERA hypothesis (“precision”).

### Subcortical–prefrontal circuit

Rhythm perception or sound envelope processing in music may engage neural activities in the subcortical–prefrontal circuit relevant for emotional processing. A primate study has shown that rhythmic drumming sounds serve as communicative signals and engage the emotional network in the subcortical areas including the amygdala and the putamen (Remedios et al., 2009). Human neuroimaging studies have shown that listening to music elicit pleasant emotion by engaging the reward system in the subcortical and cortical areas including the midbrain (e.g., ventral tegmental area, periaqueductal gray, and pedunculopontine nucleus), the nucleus accumbens, the striatum, the amygdala, the orbitofrontal cortex, and the ventral medial prefrontal cortex (Blood and Zatorre, 2001; Menon and Levitin, 2005; Salimpoor et al., 2011, 2013; Koelsch, 2014). Recent behavioral studies have also shown that listening to musical rhythm elicits positive affect and a desire to move (Zentner and Eerola, 2010; Witek et al., 2014). Similarly, perception of poetry in the presence of rhyme and regular meter lead to enhanced positive emotions, suggesting that perceiving rhythmic vocalizations may result in positive emotions (Obermeier et al., 2013). Thus, we assume that musical rhythm engages the subcortical–prefrontal circuit for emotional and reward-related processing to elicit positive affect, leading to repetition of actions to reinforce the pleasure actions (yellow arrows in Figure 2D). This meets the conditions of (3) emotion and (4) repetition in the OPERA hypothesis.

### BG-thalamo-cortical circuit

Synchronization and entrainment to a pulse in music may place high demands on information process in the BG-thalamo-cortical circuit. This notion is based on the fact that musical rhythm is more periodic while speech rhythm is quasi-periodic (Peelle and Davis, 2012). Compared with speech rhythm, musical rhythm has a more salient pulse- or beat-based timing. For example, for the phrase “happy birthday to you,” the onsets of syllable in the sound envelope are more equally time spaced in singing compared to speaking (Figure 1). Neuroimaging studies suggest that perception of beat-based timing (i.e., perception of time intervals with respect to a regular pulse) involve brain networks in the BG, the SMA, the PMC, and the DLPFC (Teki et al., 2011a,b). In fact, beat perception and synchronization increase activities in the BG, SMA, PMC, DLPFC, and STG, and enhance connectivity between the BG with the SMA, PMC, and STG (Chen et al., 2006, 2008a,b; Grahn and Brett, 2007; Grahn and Rowe, 2009; Hove et al., 2013; Kung et al., 2013). Animal studies also suggest the importance of brain networks involving the BG and cortical auditory-motor areas for beat perception and synchronization capabilities (Patel et al., 2009; Schachner et al., 2009; Hasegawa et al., 2011). In addition, tapping to a beat is associated with increased cortical responses in the DLPFC and the inferior parietal lobule (Chen et al., 2008b), which are assumed to be responsible for auditory and temporal attention (Zatorre et al., 1999; Lewis and Miall, 2003; Singh-Curry and Husain, 2009). This attention-related brain network has been shown to be more engaged in precise synchronization performance with the musical beat (Chen et al., 2008b). Taken together, synchronization and entrainment to a pulse in music engages enhanced BG-thalamo-cortical activity (light blue arrows in Figure 2D), and this fulfills the fourth and fifth conditions of the OPERA hypothesis (“repetition” and “attention”).

### Cortical motor efferent circuit

Synchronization and entrainment to a pulse in music can modulate the neural pathway for cortical motor output (red arrows in Figure 2D). Not only vocalizations but also other body movements can be synchronized and entrained to the pulse of music, such as tapping, clapping, stepping, dancing, and singing. In terms of the motor-output process in the brain, involvements of both the dorsal and ventral portions of the M1, PMC, and PFC are likely. Neuroimaging studies have shown that cortical hand motor areas are involved not only in hand motor control but also in language processing (e.g., Meister et al., 2003, 2009a,b), suggesting the importance of cortical hand motor areas for human communication. In addition, a recent transcranial magnetic stimulation (TMS) study has shown that listening to groove music modulates cortico-spinal excitability (Stupacher et al., 2013), suggesting that musical rhythm perception itself may also stimulate the motor-output pathway from the M1 to spinal cord. In sum, there are four circuits of interest in the SEP hypothesis, which may help to stimulate the brain networks underlying human communication.

## Examples of Speech and Language Disorders That Can Benefit from Rhythm-Based Therapy: Application of the “SEP” Hypothesis for Rehabilitation

The SEP hypothesis postulates that rhythm-based therapy elicits functional and structural reorganization in the neural networks for human communication in various patient populations via sound envelope processing and synchronization and entrainment to a pulse. In this section, we present examples of speech and language disorders and consider the role of rhythm in speech and language rehabilitation under the framework of the SEP hypothesis. We note, however, that the number of rhythm-based techniques currently available is very limited.

## Parkinson’s Disease

Parkinson’s disease is a neurodegenerative disorder characterized by progressive deterioration of motor function due to a loss of dopaminergic neurons in the substantia nigra (DeLong, 1990; Wichmann and DeLong, 1998; Blandini et al., 2000). In addition to the more commonly known symptoms such as muscular rigidity, tremor, and postural instability, abnormalities of voice and speech (beyond those associated with aging) are highly prevalent. Indeed, it has been estimated that over 80% of patients with PD develop voice and speech problems at some point (Ramig et al., 2008). Examples of deficits reported by clinicians include monopitch, monoloudness, hypokinetic articulation, and altered speech rate and rhythm (Darley et al., 1969). Analysis of the speech rate of patients with PD showed impaired rhythm and timing organization, such as an accelerated rate of articulation during speaking, as well as a reduction in the total number of pauses (Skodda and Schlegel, 2008; Skodda et al., 2010). When combined with the debilitating motor limb deficits, the loss of speech intelligibility and communication skills can significantly impair the quality of life of patients with PD (Streifler and Hofman, 1984).

To date, only a handful of studies have examined the speech deficits in Parkinson’s disease using neuroimaging technique (e.g., Liotti et al., 2003; Pinto et al., 2004; Rektorova et al., 2007, 2012). These studies must be interpreted with caution because the results vary depending on treatment status of patients with PD. For example, patients with PD with no medication and no deep brain stimulation showed significant dysarthria accompanying with a lack of activity in the orfacial motor cortex (M1) and cerebellum while increased activities in the PMC, SMA, and DLPFC compared with the healthy controls (Pinto et al., 2004). These abnormal cortical activities disappeared and the dysarthria symptoms improved after the deep brain stimulation of subthalamic nucleus (STN) in these patients (Pinto et al., 2004). The other fMRI studies investigated mild to moderate patients with PD with levodopa medication (Rektorova et al., 2007, 2012). Compared to healthy controls, patients with PD with the levodopa medication had increased activity in the orofacial sensorimotor cortex (Rektorova et al., 2007) and enhanced functional connectivity in the networks seeded from the periaqueductal gray matter of the midbrain (a core subcortical structure involved in human vocalization) (Rektorova et al., 2012). However, speech productions in these patients with PD with levodopa medication was comparable with that in the controls except for speech loudness, suggesting that the increased brain activity and connectivity might reflect the effects of pharmacological treatment or successful compensatory mechanisms (Rektorova et al., 2007, 2012).

Besides the deep brain stimulation and pharmacological treatments, Lee Silverman Voice Treatment (LSVT) technique has received research attention as a rehabilitation method (Ramig et al., 2001, 2004, 2007; Liotti et al., 2003; Sapir et al., 2011; Sackley et al., 2014). LSVT is designed to improve vocal function in patients with PD by enhancing loudness, intonation range, and articulatory functions. LSVT emphasizes use of loud phonation and high intensity vocal exercises to improve respiratory, laryngeal, and articulatory during speech. Compared with placebo therapy, LSVT has resulted in improvements in speech production parameters such as increases in sound pressure level (i.e., loudness) and semitone standard deviation of fundamental frequency (i.e., prosody), and these improvements were sustained even 12 months after cessation of treatment (e.g., Ramig et al., 2001). The neural correlates of LSVT have been studied by administrating levodopa medication for 4 weeks to mild and moderate patients with PD (Liotti et al., 2003). The results showed that the improvement of speech loudness following the LVST accompanied by neural activities in the striatum, insula, and DLPFC (Liotti et al., 2003).

**Figure 3:**
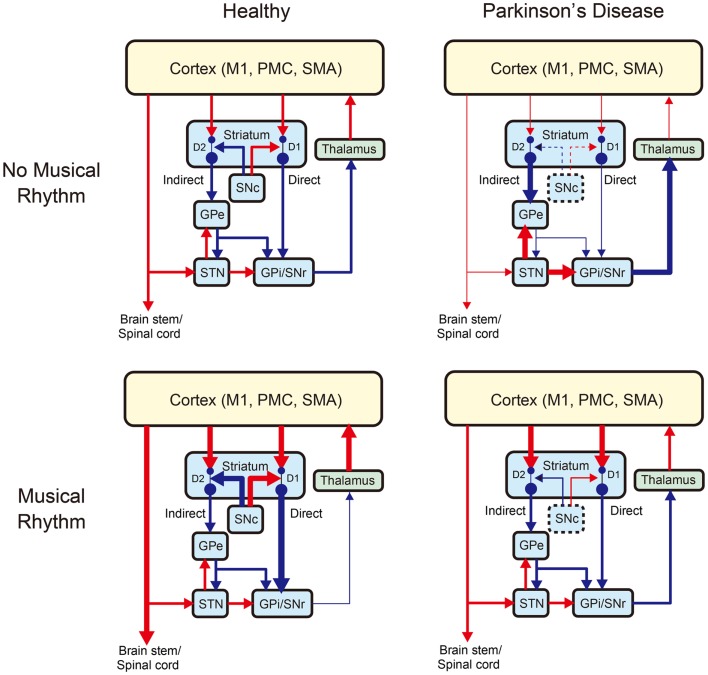
**A schematic model of changes in the basal ganglia-thalamo-cortical motor network with Parkinson’s disease and/or musical rhythm [modified from Galvan and Wichmann (2008)]**. The left and right panels show the network without and with Parkinson’s disease, while the upper and lower panels show the network without and with musical rhythm, respectively. Blue arrows indicate inhibitory projections, while red arrows indicate excitatory projections. Changes in the thickness of the arrows indicate increase (thicker arrow) or decrease (thinner arrow) of the projections relative to the normal situation. The dashed lines around the SNc (substantia nigra pars compacta) indicate degeneration of dopaminergic neurons caused by Parkinson’s disease. GPe, external segment of globus pallidus; GPi, internal segment of globus pallidus; SNr, substantia nigra pars reticulata; STN, subthalamic nucleus; M1, primary motor cortex; PMC, premotor cortex; and SMA, supplementary motor area. Striatum has “direct” and “indirect” pathways to the basal ganglia (BG) output nuclei (GPi/SNr). Light blue, green, and yellow colors denote BG, thalamus, and cortex, respectively. D1 and D2 indicate subtypes of dopamine receptor. Parkinson’s disease induces hyper-activation of BG output nuclei (GPi/SNr) inhibiting activities of the thalamocortical projection neurons, which in turn decreases motor output, while musical rhythm induces hypo-activation BG output nuclei and thereby facilitates thalamo-cortical motor output.

Under the SEP hypothesis, patients with PD can benefit from synchronization and entrainment to a pulse in music to stimulate the subcortical–prefrontal network and the BG-thalamo-cortical network. As illustrated in Figure 3, the BG-thalamo-cortical network functions normally in healthy individuals (top left panel), whereas the network becomes abnormal in PD because of the degeneration of the dopamine-producing neurons in the substantia nigra pars compacta (SNc) (top right panel). The projections from the SNc to the striatum regulates the cortico-strital projections, and if the dopamigeneric neurons in the SNc are depleted, it leads to reduced inhibition in the “direct” pathway to the BG output nuclei (i.e., GPi: internal segment of globus pallidus and SNr: substantia nigra pars reticulata), which carries dopamine “D1” receptors. The degeneration of dopamigeneric neurons in the SNc also leads to increased inhibition in the “indirect” pathway to the BG output nuclei (GPi/SNr) via external segment of globus pallidus (GPe) and STN carrying dopamine “D2” receptors. Net action of the degeneration of dopanigeneric neurons in the SNc leads to the hyper-activation of the BG output nuclei (GPi/SNr) inhibiting activities of thalamocortical projection neurons, which in turn negatively affects motor output [for more detail, see DeLong (1990), Wichmann and DeLong (1996), Blandini et al. (2000), Galvan and Wichmann (2008), and Smith et al. (2012)].

In this model, we assume that pleasurable musical rhythm induces increased endogenous dopamine release in the striatum (bottom left panel in Figure 3). This is based on a previous study, which showed an increase in dopamine release and hemodynamic response in the striatum during listening pleasurable music (Salimpoor et al., 2011). In our model, the increased dopamine release leads to increased inhibition in the BG output neuclei (GPi/SNr) and thereby facilitates thalamo-cortical motor output and a desire to move. This idea is also supported by the other studies that showed modulation in the cortico-spinal excitability (Stupacher et al., 2013) and increased activity and connectivity in the brain network including the striatum, PMC, and SMA during perceiving and synchronizing with the musical beats (Chen et al., 2006, 2008a,b; Grahn and Brett, 2007; Grahn and Rowe, 2009; Kung et al., 2013). We also postulate that pleasurable musical rhythm may increase dopamine release even in patients with PD considering that some of the GNc neurons (approximately 30–50%) remain intact even at the time of death (e.g., Davie, 2008). Indeed, a number of studies have shown improvements of motor function in patients with PD during rhythmic auditory stimulation (RAS) (e.g., Thaut et al., 1996; McIntosh et al., 1997; de Bruin et al., 2010; Nombela et al., 2013), suggesting that musical rhythm may facilitate thalamo-cortical motor output in patients with PD (bottom right panel in Figure 3).

If our model is correct, then musical rhythm may reduce reliance on levodopa medication and/or deep brain stimulation. However, there remain a few untested assumptions. For example, patients with PD show impaired emotional recognition in music (e.g., van Tricht et al., 2010), suggesting that the ventral portion of the striatum is also affected in patients with PD. Therefore, dopamine release in the striatum may not be increased by music in patients with PD as seen in healthy individuals. Positron emission tomography (PET) can be used to test this hypothesis (Laruelle, 2000; Salimpoor et al., 2011), and whether this may be affected by intervention. A recent study has shown improved perceptual and motor timing in patients with PD after a 4-week music training program with rhythmic auditory cueing (Benoit et al., 2014), suggesting a possible benefit of RAS on the treatment of PD. However, the participants of that study consisted only of mild to moderate patients with PD (Benoit et al., 2014). Future studies will need to clarify whether the RAS is also beneficial for severe patients with PD, and how the therapeutic effect is different. It may also be important to test whether RAS (simple metronome stimulation as well as rhythmic musical stimulation) improves speech function in patients with PD, given gait functions have been a topic of research interest (e.g., Thaut et al., 1996; McIntosh et al., 1997; de Bruin et al., 2010; Nombela et al., 2013). Additional neuroimaging studies are required to examine the brain networks underlying speech and hand/foot motor processes in patients with PD.

## Stuttering

Stuttering is a developmental condition that affects fluency of speech. It begins during the first few years of life, and affects approximately 5% of preschool-aged children (Bloodstein, 1995). Symptoms include repetition of words or parts of words, as well as prolongations of speech sounds, resulting in disruptions in the normal flow of speech.

As illustrated in Figure 2C, during normal speech production, the left IFG and PMC projects to the M1 for vocal-motor output and the right IFG and PMC are monitoring sensory feedback together with the temporal cortex and the cerebellum (blue arrows). However, individuals who stutter show abnormalities in the left IFG and PMC and compensatory hyperactivity in the right IFG and the cerebellum (Fox et al., 1996; Brown et al., 2005; Kell et al., 2009), suggesting that stuttering is associated with poor feed-forward motor command and excessive reliance on auditory feedback control (Max et al., 2004; Civier et al., 2010, 2013). In addition, stuttering is associated with reduced activity and connectivity in the brain network including the BG and the SMA (e.g., Toyomura et al., 2011; Chang and Zhu, 2013), suggesting that stuttering may be due to dysfunction in the BG-thalamo-cortical circuit to produce timing cues for the initiation of the next motor segment in speech (Alm, 2004).

To date, examples of fluency shaping methods for stuttering include altered auditory feedback (e.g., Hargrave et al., 1994; Ryan and Van Kirk Ryan, 1995; Stuart et al., 1996; Armson and Kiefte, 2008), prolonged speech that uses slow and exaggerated speech production (e.g., O’Brian et al., 2010), training of oral-motor co-ordination (e.g., Riley and Ingham, 2000), and the Lidcombe technique, a response-contingent program that involves parents to shape the child’s utterances (e.g., Lattermann et al., 2008). The altered auditory feedback methods are considered to be effective to change the excessive reliance on auditory feedback control, while the other speech production trainings would help to reform feed-forward speech commands. Yet, a therapy that focuses specifically on stimulating the BG-thalamo-cortical circuit to enhance rhythmic speech production would also be warranted (Alm, 2004).

The SEP hypothesis assumes that individuals who stutter may benefit from rhythm-based therapy using synchronization and entrainment to a pulse for stimulating the BG-thalamo-cortical circuit. Behavioral studies support this notion by showing that the presence of rhythmic auditory signals such as metronome beats, when synchronized with speech production, induces strong fluency-enhancing effects in individuals who stutter (e.g., Brady, 1969, 1971). A recent fMRI study also supports this notion by showing that BG activities of stuttering speakers increased to the level of normal speech controls when speaking with the metronome beats (Toyomura et al., 2011).

Nevertheless, given that stuttering is a relapse-prone disorder (Craig, 2002), long-term management strategies are likely to be useful when dealing with this disorder over a lifetime. Accordingly, future studies need to test how long the metronome-induced fluency sustains after removing the rhythmic sounds. It has been suggested that the BG-thalamo-SMA circuit is dominant for self-initiation of speech, while the PMC-thalamo-cerebellar circuit is dominant for externally cued speech (Alm, 2004). Therefore, one of the challenges for future studies is to transition from the externally cued (PMC-centered) speech to self-initiated (SMA-centered) speech in the treatments using metronome-guided cues. To engage the BG-thalamo-SMA circuit, it may be useful to use non-isochronous metronome stimuli to promote the patients to find a pulse and initiate rhythmic speech by themselves. In addition, future studies need to test whether structural and functional reorganization occur in the BG-thalamo-cortical circuit after the intervention using the rhythmic auditory cues. Concurrently, more basic studies are needed to clarify whether the neural underpinnings of stuttering overlap with those of rhythm processing in music. Investigations of the abilities of musical rhythm processing in individuals who stutter using an amusia battery (e.g., Peretz et al., 2003; Fujii and Schlaug, 2013) may help further our understanding of possible overlapping brain networks. In addition, there is a need to test whether synchronization to a pulse in music have the similar fluency-enhancing effect for stuttering speakers compared with the synchronization to a metronome.

## Aphasia

Aphasia is a common and devastating consequence of stroke or other brain injuries that results in language-related dysfunction. When speech production is impaired, the patients are broadly classified into the category of “non-fluent aphasia.” In such cases, a lesion in the left posterior frontal region (Broca’s area) is often observed. Many patients with large left hemisphere lesions have poor prognosis, despite having received years of intensive speech therapy (Lazar et al., 2010). However, emerging evidence suggests that some techniques have the potential to improve the verbal communication skills of these patients, as well as to reorganize the underlying neural processes related to language. For example, inspired by the clinical observation that patients with non-fluent aphasia can sing words even though they are unable to speak (Gerstmann, 1964; Yamadori et al., 1977), melodic intonation therapy (MIT) has received much research attention over the past few years. The main components of this speech therapy technique are (1) melodic intonation, (2) the use of formulaic phrases and sentences, and (3) slow and periodic verbalization with left-hand tapping (Schlaug et al., 2008, 2010). Within a therapy session, the therapist instructs the patient to intone (or “sing”) simple phrases while slowly tapping their left hand with each syllable.

Emerging evidence involving open-label studies has revealed some positive treatment effects (Wilson et al., 2006; Schlaug et al., 2008; Wan et al., 2014). However, the question of “why” MIT works remains the subject of intense debate. The contribution of singing is supported by the neuroimaging findings of right hemisphere lateralization of singing processing when compared to speaking (e.g., Ozdemir et al., 2006), as well as by studies showing reorganization of right hemisphere structure and function following therapy (e.g., Schlaug et al., 2008). However, it is important to note that the latter studies often included patients with very large lesions that sometimes cover most of the left hemisphere, thus precluding analysis of language-related areas within that hemisphere. In addition, although melodic intonation is usually emphasized as a major difference of singing compared with speaking, the difference between singing rhythm and speaking rhythm has been overlooked (see Figure 1 as an example).

Recent studies have highlighted the potential role of rhythm in aphasia treatment. For example, aphasia recovery, as denoted by correct syllable production, was examined by comparing singing therapy, rhythmic therapy, and standard speech therapy (Stahl et al., 2011, 2013). The results showed that, when compared to singing therapy, the rhythmic therapy was similarly effective (Stahl et al., 2011, 2013). Moreover, patients with lesions that cover the BG were found to be highly dependent on the external rhythmic cues (Stahl et al., 2011). Taken together, this study highlights the role of rhythm in aphasia recovery.

The SEP hypothesis postulates that the rhythmic components (e.g., singing rhythm, left-hand tapping) of MIT can help to facilitate sound envelope processing and synchronization and entrainment to a pulse. That is, the predictability of formulaic phrases and sentences requires precise encoding of pulse or periodic timing of vocalizations, while left-hand tapping can facilitate synchronization and entrainment to the pulse. Thus, under the SEP framework, MIT may be interpreted as an effective way to engage (a) the auditory afferent circuit to encourage precise encoding of sounds, (b) the subcortical–prefrontal circuit to motivate patients, (c) the BG-thalamo-cortical circuit to facilitate beat-based timing process, and (d) the efferent cortical motor circuit to promote the motor output (Figure 2D).

A rationale for MIT is the potential to engage and unmask language-capable regions in the unaffected right hemisphere such as the structural reorganization of arcuate fasciculus, a fiber bundle connecting the posterior superior temporal region and the posterior inferior frontal region (Schlaug et al., 2008, 2010; Wan et al., 2014). The SEP hypothesis assumes that sound envelope processing may be lateralized to the temporal cortex in the right hemisphere (Figure 2B). If MIT engages high demands on the right temporal cortex to encode sound envelope precisely, it may also increase the connectivity from the right temporal cortex to the right inferior frontal gyrus (IFG). Importantly, rhythmic therapy in aphasia patients with left basal ganglia lesion resulted in improved production of common formulaic phrases that are known to be supported by right BG-thalamo-cortical network (Stahl et al., 2013), suggesting that rhythm therapy for aphasia might also induce alterations in the right BG-thalamo-cortical network. The left-hand tapping in MIT might be also interpreted as a way to recruit enlarged involvement of contralateral right motor areas (i.e., dorsal and ventral portions of the right M1, PMC, and PFC) and thereby facilitate motor output of the unaffected hemisphere.

## Autism

One of the core features of autism spectrum disorder (ASD) is impairment in language and communication. For children with ASD, the ability to speak early is associated with improved quality of life. Research has reported the presence of motor and oral-motor impairments in ASD children who have expressive language deficits (Belmonte et al., 2013; McCleery et al., 2013).

To date, very few interventions have specifically targeted the oral-motor aspects in ASD. One is the prompts for restructuring oral muscular phonetic targets (PROMPT) model, which is a play-based technique that involves vocal modeling and physical manipulations of the children’s oral-motor system to facilitate the production of a speech target (Chumpelik, 1984). A pilot study reported speech improvements in five non-verbal children with ASD after receiving PROMPT intervention (Rogers et al., 2006). Another therapy technique that incorporates a motor component is auditory-motor mapping training (AMMT), which is an active multisensory therapy designed to facilitate speech output in completely non-verbal children with autism (Wan et al., 2010). This technique aims to promote speech production directly by training the association between speech sounds and articulatory actions using slow and melodic intonating vocalizations with bimanual motor activities (Wan et al., 2010). While some of the components of AMMT overlap with those of MIT in phasia, a unique aspect of AMMT is the use of a set of tuned drums and bimanual motor actions to facilitate sound-motor mapping. An initial proof-of-concept study indicated the therapeutic potential of AMMT in facilitating speech development in autism (Wan et al., 2011).

Similar to MIT in aphasia, we assume that AMMT can be categorized into non-rhythmic and rhythmic components. The former relates to intoned vocalizations, and the latter relates to spoken syllables being linked with the bimanual motor actions on the tuned drums. Under the SEP framework, the rhythmic component in AMMT can be useful in a number of ways. First, perception of rhythmic drumming and vocal sounds may stimulate the auditory afferent circuit for the precise encoding of sound envelope or temporal events. Indeed, it has been shown that ASD is associated with developmental abnormalities in the brainstem and cerebellum *in utero*, which can lead to abnormal timing and sensory perception in ASD (Trevarthen and Delafield-Butt, 2013). Second, synchronization and entrainment of rhythmic vocalizations and bimanual motor actions may be effective to stimulate the speech motor and language networks in ASD. In the DIVA model, the left vPMC and pIFG are involved in both speech production and sensorimotor mapping and have functional correspondence to the mirror-neuron system (Rizzolatti et al., 1996; Kohler et al., 2002; Guenther et al., 2006; Tourville and Guenther, 2011). A number of neuroimaging studies have suggested that ASD is associated with abnormalities in the IFG and posterior superior temporal sulcus (pSTG) (Herbert et al., 2002; De Fosse et al., 2004; Kleinhans et al., 2008; Mengotti et al., 2011; McCleery et al., 2013). Moreover, another neuroimaging study suggests that motor dysfunction in ASD is associated with abnormality in BG-thalamo-SMA circuit (Enticott et al., 2009). Thus, synchronization and entrainment of rhythmic vocalizations and bimanual motor actions in AMMT may help ameliorate speech production and sensorimotor mapping deficits in ASD by engaging the BG-thalamo-cortical (SMA, PMC, IFG, and STG) circuit.

## Conclusion

In this paper, we consider the role of rhythm in speech and language rehabilitation. The emerging research field of music and neuroscience led us to propose the SEP hypothesis, which postulates that (1) sound envelope processing and (2) synchronization and entrainment to a pulse, may help to stimulate brain networks for human communication. Within the SEP framework, we present four possible circuits that may help to stimulate the brain networks underlying human communication: (i) the auditory afferent circuit consisted of brainstem, thalamus, cerebellum, and temporal cortex for precise encoding of sound envelope and temporal events; (ii) the subcortical–prefrontal circuit for emotional and reward-related processing; (iii) the BG-thalamo-cortical circuit for processing beat-based timing; and (iv) the cortical motor efferent circuit for motor output. We hope that future studies combining neuroimaging techniques and randomized control designs with the SEP framework will help to evaluate the efficacy of the rhythm-based therapies.

## Conflict of Interest Statement

The authors declare that the research was conducted in the absence of any commercial or financial relationships that could be construed as a potential conflict of interest.
